# The Effect of Different Mixing Methods on the Flow Rate and Compressive Strength of Mineral Trioxide Aggregate and Calcium-Enriched Mixture

**Published:** 2014-12-24

**Authors:** Shahriar Shahi, Negin Ghasemi, Saeed Rahimi, Hamid Reza Yavari, Mohammad Samiei, Maryam Janani, Mahmood Bahari, Sanaz Moheb

**Affiliations:** a *Dental and periodontal research center, Dental School, Tabriz University of Medical Sciences, Tabriz, Iran;*; b * Department of Endodontics, Dental School, Tabriz University of Medical Sciences, Tabriz, Iran; *; c * Department of Operative Dentistry, Dental School, Tabriz University of Medical Sciences, Tabriz, Iran; *; d * Private Practice, Tabriz, Iran*

**Keywords:** Calcium-Enriched Mixture, CEM, Compressive Strength, Flow, Mineral Trioxide Aggregate, MTA

## Abstract

**Introduction: **Flow rate (FR) and compressive strength (CS) are important properties of endodontic biomaterials that may be affected by various mixing methods. The aim of this experimental study was to evaluate the effect of different mixing methods on these properties of mineral trioxide aggregate (MTA) and calcium-enriched mixture (CEM) cement. **Materials and methods: **Hand, amalgamator and ultrasonic techniques were used to mix both biomaterials. Then 0.5 mL of each mixture was placed on a glass slab to measure FR. The second glass slab (100 g) was placed on the samples and 180 sec after the initiation of mixing a 100-g force was applied on it for 10 min. After 10 min, the load was removed, and the minimum and maximum diameters of the sample disks were measured. To measure the CS, 6 sample of each group were placed in steel molds and were then stored in distilled water for 21 h and 21 days. Afterwards, the CS test was performed. Data were analyzed with multi-variant ANOVA and post hoc Tukey tests. The level of significance was set at 0.05.** Results: **There were significant differences in FR of MTA and CEM cement with different mixing techniques (*P*<0.05). In the MTA group, none of the mixing techniques exhibited a significant effect on CS (*P*>0.05); however, in CEM group the CS at 21-h and 21-day intervals was higher with the hand technique (*P*<0.05).** Conclusion**: Mixing methods affected the flowability of both biomaterials and compressive strength of CEM cement.

## Introduction

Compressive strength (CS) and an increase in the strength of a given biomaterial over time is an indicator of setting reaction and stability of the material [1, 2]. As root-end filling materials, mineral trioxide aggregate (MTA) and calcium-enriched mixture (CEM) cement must bear mastication forces and CS is one of the properties that enables them to do so. MTA is mixture of tricalcium aluminate, dicalcium silicate, tricalcium silicate, tetracalcium alumino ferrite, and bismuth oxide [3, 4]. It is used in perforation repair procedures and periapical surgery because of induction of regeneration in the periradicular area, provision of a proper seal between the root canal and the external surface of the root, setting in the presence of blood, and its biocompatibility. However, it has some disadvantages such as long setting time and handling difficulty [5-9]. In spite of the 4-h setting time for MTA, it takes few days for its CS to reach the maximum level; with primary CS of 40 MPa after mixing rising up to 67 MPa within 21 days [10]. This CS is higher than Portland cement [11]. 

On the other hand the main components of CEM cement are metallic oxides and hydroxides, calcium phosphate and calcium silicate [6-12]. The clinical applications of CEM are similar to MTA but it does not have the notable disadvantages of MTA, such as long setting time and difficult handling [5, 13, 14]. For the CEM cement there is no published data on its CS. 

Flow rate (FR) is another important physical property of dental biomaterials; poor FR of MTA has been reported as one of its disadvantages [10]. One study has reported superior FR of CEM cement compared to MTA [5].

The physical and chemical properties of dental materials are influenced by the mixing technique. Mechanical mixing (trituration) can decrease the number of air-filled spaces between the particles, thus leading to an increase in the wetted surfaces, which improves the uniformity of the paste [15]. Ultrasonic energy, another mixing technique, can disperse the particles which are usually located next to each other in clusters; therefore, the overall reaction surface increases [16]. Based on a previous reported studies, ultrasonic energy increase the CS and tensile strength and also density of the materials, and tends to decrease the setting time and improve the handling properties [17]. 

The present study was designed to evaluate the effect of mixing methods on the FR and CS of MTA and CEM cement.

## Materials and Methods

***Preparation of the samples***

This study was conducted on six samples of each material with three different mixing techniques (total number of 18 samples). Before mixing, White ProRoot MTA (Dentsply, Tulsa Dental, Tulsa, OK, USA), CEM cement (BioniqueDent, Tehran, Iran) along with the mixing pads, spatulas and the glass slabs, were placed in incubator with 37±1^°^C temperature for 1 h. The powder (1 g) and liquid (0.34 g) of test materials were mixed using hand mixing, amalgamator and ultrasonic techniques. In the conventional hand mixing technique, mixing was done according to the manufacturers’ instructions. In the ultrasonic technique, the method was exactly the same as hand mixing, except for handling the mixture with an ultrasonic scaling tip (Juya Electronic Co., Tehran, Iran). In the amalgamator technique, appropriate amounts of the materials (1 g powder and 0.34 g liquid, as ordered by the manufacturers) were placed in the mixing chamber of an amalgamator (Promix TM; Dentsply Caulk, York, PA, USA) and mixed for 20 sec.

**Table 1:** The mean (SD) of flow rates (mm)

**Mixing technique**	**Material (N)**	**Mean (SD)**	***P-*** **Value**
**Hand**	CEM cement (6)	12.27 (0.52)	0.001
	MTA (6)	11.27 (0.37)	
**Amalgamator**	CEM cement (6)	11.45 (0.19)	0.07
	MTA (6)	11.88 (0.31)	
**Ultrasonic**	CEM cement (6)	12.48 (0.56)	0.001
	MTA (6)	11.75 (0.21)	

***Determination of flowability***

The FR of the materials was tested according to the ISO 6876 criteria [18]; 2 mL of mixed paste was placed on the center of a glass plate (with dimensions of 40×40×5 mm^3^ and 20 g weight). After 3 min, another 100-g glass plate was placed on top of the material. The load was removed 10 min after the start of mixing, and the minimum and maximum diameters of the sample disks were measured by a digital caliper (Cole-Parmer Canada Inc, Montreal, Canada) with a resolution of 0.01 mm; then the mean values were calculated. If the disks were not uniformly circular (the maximum and minimum diameters were not within 1 mm), the test was repeated. 

***Determination of compressive strength***

CS of the samples was measured using ISO 6876 guidelines [18]. Sufficient amounts of each material were mixed with the three techniques, and were the packed in steel molds (measuring 12 mm in height and 6 mm in diameter) within 2 min after initiation of mixing. Three min after mixing, the whole sets (molds and the samples) were placed in an incubator at 37±1^°^C and 100% relative humidity for 3 h. Six samples from each mixing technique (18 samples on the whole) from each material, which had no defects or bubbles, were selected for each time interval (21 h and 21 days). The samples were stored in distilled water for 21 h or 21 days and then underwent a CS test in a universal testing machine (Hounsfield Test Equipment, model: H5K-S, Perrywood Business Park, Honey Corckland, Salfords, Redhill, Surrey, UK) at a crosshead speed of 0.5 mm/min. The CS was calculated in MPa using the following formula: CS=4*p/µd*^2^ where *p* is the maximum force applied in Newtons, and *d* is the mean diameter of the specimen in mm.

***Analysis of data***

Multivariate analysis of variance was used to evaluate the significant effect of the material type, time intervals, and mixing methods. The post hoc Tukey test was used for the two-by-two comparison of the groups. SPSS software (SPSS version 18.0, SPSS, Chicago, IL, USA) was used for the analysis of data. The level of statistical significance was defined at 0.05.

## Results

The FR of the test materials had significant differences with different mixing techniques (*P*=0.001 for MTA and *P*=0.004 for CEM cement). CEM cement exhibited significantly the least FR with the amalgamator technique. However, there were no significant differences between the hand and ultrasonic mixing techniques (*P*=0.9). For MTA, the amalgamator and hand techniques exhibited the highest and lowest FR, respectively (*P*<0.05). The mean FR of the test materials is presented in Table 1. 

Tables 2 and 3 represent the mean CS values of the materials. In MTA samples, the effect of different mixing techniques on compressive strength was not significant at any time (*P*=0.09 and *P*=0.1 for 21-h and 21-day intervals, respectively). In contrast, CEM exhibited statistically significant differences in the CS with different mixing techniques at both time intervals, with the highest CS values belonging to hand technique at 21-h and 21-day intervals (*P*=0.02 for 21-h and *P*=0.01 for 21-day samples, respectively); however, the two other techniques were not significantly different from each other (*P*=0.08 for 21-h interval and *P*=0.1 for 21-day interval).

## Discussion

In the present study, the effects of three mixing techniques (hand mixing, amalgamator and ultrasonic mixing) on the FR and CS of CEM cement and MTA were evaluated and compared. 

Regarding the CS in CEM cement samples, there were differences among the different mixing techniques at 21-h interval, with significantly higher CS values belonging to samples prepared with hand mixing technique. In other words, it can be claimed that it is advisable to mix CEM cement with the hand technique to achieve the best CS values because such results were repeated with the 21-day samples, as well. In MTA group, the CS with the ultrasonic technique was not significantly different compared to hand mixing technique, which is contrary to the results reported by Basturk *et al.* [15]. It might be attributed to the time difference for the test. In their study, the CS tests were carried out 4 days after mixing the cement [15]. Based on the results of the present study, regarding CS in CEM cement samples, there were differences between the different mixing techniques at 21-h interval, with significantly higher CS related to hand mixing technique. 

In order to achieve optimal properties of hydraulic cements, the powder particles should be completely mixed with liquid. The hand-mixed cements are weak; because air is trapped between the particles; however, encapsulated cements are strong and encapsulation during mechanical mixing can save time and result in a homogeneous mixture with favorable physical properties [15]. Nekoofar *et al.* [16] compared the ultrasonic and hand mixing and showed that ultrasonic energy results in higher surface hardness of MTA. In addition, a study showed higher CS for MTA with mechanical mixing compared to hand mixing [15]. The CS of hydraulic cements is an indication of progression of hydration reaction and is a reflection of the setting process of these materials [19]. CS is not critical for root-end filling materials because they are not subjected to direct occlusal forces; however, this parameter is very important for coronal restorations, such as the use of biomaterials in repairing furcal restorations or apexogenesis of the teeth with immature apices [12, 20]. The CS of MTA is influenced by the material type, the liquid mixed with it, the pressure applied, the acid etching process, mixing technique and the storage conditions [2, 21]. 

**Table 2:** Mean (SD) of compressive strength (MPa) after 21 h

**Mixing technique**	**Material (N)**	**Mean (SD)**	***P*** **-value**
**Hand **	CEM cement (6)	257.33 (20.53)	0.001
	MTA (6)	151.33 (11.02	
**Amalgamator **	CEM cement	211.50 (12.82)	0.12
	MTA (6)	156.78 (15.10)	
**Ultrasonic **	CEM cement (6)	221.67 (28.43)	0.02
	MTA (6)	150.65 (21.52)	

There are no published data available on the CS of CEM cement. Based on the results of present study, irrespective of the differences in mixing techniques, the CS of CEM cement at 21-day interval is similar to MTA. However, contrary to MTA, no significant increase was observed in the CS of CEM cement samples from 21-h to 21-day intervals, which might be attributed to the faster setting of CEM cement. The size of the particles [12, 17] is one of the most important factors affecting the hydration rate and setting properties of materials and based on the studies carried out to date, the particle sizes of CEM are smaller than MTA, resulting in an increase in contact surface area with the liquid and improvement in strength and handling properties [5]. 

MTA is predominantly a mixture of dicalcium silicate and tricalcium silicate and since the hydration rate of dicalcium silicate is slower; hydration process and setting which influence the strength, are longer [3]. An important consideration regarding CEM cement is gaining the highest CS with hand mixing method which might be explained by the fact that small particle size and proper hydration may resulted in better findings.

The other variable of the present study was the FR of the materials with different mixing techniques. The FR of CEM cement was similar in hand and ultrasonic mixing techniques and higher than amalgamator method. MTA had the least FR with hand method; followed by ultrasonic and amalgamator techniques. The FR of MTA with the amalgamator was better than CEM. In addition, regarding the flow test of CEM cement mixed with the amalgamator technique, care should be taken because based on the results reported by Asgary *et al.* [5], CEM cement showed higher FR compared to MTA except for mixing with an amalgamator.

**Table 3:** Mean (SD) of compressive strength (MPa) after 21 days

**Mixing technique**	**Material (N)**	**21-day strength**	***P*** **-value**
**Hand **	CEM cement (6)	267.67 (21.96)	0.001
	MTA (6)	257.00 (13.45)	
**Amalgamator **	CEM cement	238.33 (10.41)	0.001
	MTA (6)	249.33 (16.92)	
**Ultrasonic **	CEM cement (6)	248.33 (24.19)	0.003
	MTA (6)	260.33 (23.71)	

## Conclusion

Compressive strength of CEM cement is affected by mixing technique but the same scenario does not apply for MTA. Also, it can be concluded that the flow of CEM cement and MTA is not affected by mixing procedures. The mixing technique and the time elapsed after placement of the material in the clinical settings should always be considered by the clinician to achieve the best physical properties.
